# Depression and cancer-related fatigue in elderly colorectal cancer patients: A cross-sectional study with focus on the mediating role of chemotherapy-induced peripheral neuropathy and the moderating role of perceived social support

**DOI:** 10.1097/MD.0000000000046162

**Published:** 2025-11-21

**Authors:** Wenjing Xu, Qian Cao, Chen Xu, Jinyou Song, Yuling Yang, Ying Chen

**Affiliations:** a Department of Oncology, Affiliated Hospital of Jiangnan University, Wuxi, Jiangsu Province, China; b Wuxi Medical School, Jiangnan University, Wuxi, Jiangsu Province, China.

**Keywords:** cancer-related fatigue, chemotherapy-induced peripheral neuropathy, depression, elderly colorectal cancer patients, moderated mediation model, perceived social support

## Abstract

This study examines whether chemotherapy-induced peripheral neuropathy (CIPN) mediates the relationship between depression and cancer-related fatigue (CRF) and further investigates the moderating role of perceived social support (PSS) in this relationship among Chinese older adults with colorectal cancer (CRC). A total of 290 elderly Chinese patients with CRC undergoing chemotherapy were recruited. Structured questionnaires were administered to assess levels of depression, symptoms of CIPN, CRF, and PSS. Moderated mediation models were tested using the PROCESS macro (Models 4 and 8) in SPSS, controlling for relevant demographic and clinical covariates, to examine the hypothesized path relationships and interaction effects. After controlling for relevant covariates, CIPN was found to partially mediate the relationship between depression and CRF (indirect effect = 0.231), accounting for 38.44% of the total effect. Moreover, PSS significantly moderated the associations between depression with both CIPN (β = 0.101, *P* < .05) and CRF (β = 0.018, *P* < .05), such that higher levels of PSS strengthened these relationships. CIPN may be a potential pathway reflecting how emotional disturbances, particularly depression, are associated with CRF in elderly patients with CRC. The subjective experience of CIPN appears to be closely related to depressive symptoms. Contrary to the classical stress-buffering hypothesis, PSS did not exhibit a protective effect; rather, in certain cultural contexts, it may be linked to the physiological manifestations of negative emotions. These findings underscore the need for culturally sensitive and context-specific approaches to emotional intervention and social support in symptom management to optimize individualized care strategies for elderly CRC patients.

## 1. Introduction

Colorectal cancer (CRC) is the third most common malignant tumor globally,^[1]^ and the second most common in China, with elderly adults accounting for more than 60% of cases.^[2]^ In elderly CRC patients, treatment is often complicated not only by the physical burden of disease but also by age-related declines in cognitive and functional capacities, along with relatively limited psychosocial resources, all of which further exacerbate symptom experiences and pose challenges to effective management.^[3]^

Oxaliplatin-based chemotherapy remains the standard treatment for elderly patients with CRC. However, it is frequently accompanied by notable toxic side effects, most prominently chemotherapy-induced peripheral neuropathy (CIPN),^[4]^ which manifests as numbness, tingling, sensory loss, or even motor dysfunction in the extremities. CIPN is particularly common and persistent in older adults, substantially impairing daily functioning and treatment adherence.^[5]^ Cancer-related fatigue (CRF), another prevalent symptom in this population,^[6]^ is characterized by a persistent sense of exhaustion and reduced energy that is not alleviated by rest. CRF significantly compromises patients’ quality of life.^[6]^ Although current non-pharmacological interventions such as physical activity, psychosocial support, and lifestyle modification have been recommended,^[7,8]^ their effects remain limited. The underlying mechanisms of CRF and effective intervention strategies still require further investigation.

Among tumor-related symptoms, emotional factors, particularly depressive states, have been shown to be strongly associated with several subjective symptoms. Depressive states may not only directly exacerbate fatigue,^[9,10]^ but may also amplify the experience of subjective symptoms such as pain, numbness, and fatigue by enhancing attention to physical discomfort.^[8,11]^ Hsiao et al.^[12]^ demonstrated that depression can indirectly contribute to fatigue through subjective symptoms such as pain, suggesting that subjective symptomatology may serve as a mediating link between emotional states and functional outcomes. CIPN as a toxic response dominated by subjective feelings, may be susceptible to modulation by emotional states. In addition, CIPN often triggers exercise avoidance behavior,^[5]^ despite physical activity being one of the most effective non-pharmacological strategies to alleviate CRF.^[7]^ These findings suggest that depression may be associated with heightened the perceptions of CIPN, which in turn promotes behavioral avoidance and subsequently exacerbates CRF. However, empirical evidence supporting this mechanism remains limited, particularly in elderly Chinese patients with colorectal cancer, and has yet to be systematically investigated.

Perceived Social Support (PSS) is widely recognized as a key moderator of negative emotions on health.^[13]^ According to the Stress-buffering model, PSS can reduce the negative effects of emotions on physiological outcomes to a certain extent by reducing the perception of stress and enhancing coping skills.^[14]^ However, the mechanism of action of social support has a dual dependence on cultural and subjective constructs. It has been shown that the mechanism of action of social support is profoundly influenced by the context of cultural, social, and individual beliefs, and that the meaning and acceptance of support in different cultures significantly alters the efficacy of its action on psychological and physiological health.^[15]^ However, no study has explored how PSS may be associated with the potential links between depression, CIPN, CRF, especially in culturally sensitive elderly cancer patients.

Based on this, the present study constructed a moderating mediator model with elderly CRC chemotherapy patients to explore whether depression indirectly relates to fatigue levels through CIPN symptoms, and to further test whether PSS plays a moderating role in this pathway. Through this model, we aim to reveal the interaction mechanism between mood-symptoms-social resources, and provide preliminary theoretical insight and empirical reference for culturally sensitive intervention strategies.

## 2. Methods

### 2.1. Study setting and sampling

This study was a cross-sectional study involving 12 independent variables. In terms of sample size estimation, based on the conventional recommendation of 5 to 10 samples per independent variable and considering a 20% invalid response rate,^[16]^ the initial required sample size was calculated to be between 72 and 144 cases. However, since a mediation model was planned to be constructed in this study, a sample size of at least 200 cases was deemed necessary to ensure the stability of the model testing.^[17]^ Therefore, the final sample size was determined to exceed 200 cases, with 290 elderly colorectal cancer patients recruited through convenience sampling from the Affiliated Hospital of Jiangnan University from January 2024 to March 2025. After data collection, a post hoc power analysis of the moderated mediation model paths was conducted using G-Power 3.1 software. The results showed that the statistical power for all paths exceeded 0.8, indicating that the current sample of 290 cases was sufficient to detect the true effects of each path.

Inclusion criteria age ≥ 60 years, pathologically confirmed colorectal cancer; receiving or recently receiving oxaliplatin-containing chemotherapy regimen; reported at least one symptom on the symptom experience section of the Chinese version of the chemotherapy-induced peripheral neuropathy assessment tool,^[18]^ with a score ≥ 1; had basic communication skills and sufficient cognitive ability to understand and independently complete the questionnaire; and provided written informed consent and voluntarily agreed to participate in the study.

The exclusion criteria were presence of comorbidities that may cause peripheral neuropathy (such as diabetes mellitus, post-stroke sequelae, Parkinson disease, etc); presence of significant psychiatric or cognitive disorders (such as Alzheimer disease, severe cognitive impairment, etc); severe hepatic, renal, or cardiac dysfunction; prior exposure to other neurotoxic chemotherapeutic agents apart from oxaliplatin (e.g., taxanes); and refusal to participate or withdrawal during the study.

### 2.2. Ethical considerations

This study was approved by the Ethics Committee of the Affiliated Hospital of Jiangnan University (Approval No. LS2023101) and registered in the Chinese Clinical Trial Registry (Registration No. ChiCTR2400079958). The study was conducted in strict accordance with the ethical principles outlined in the Declaration of Helsinki. Written informed consent was obtained from all participants prior to enrollment, with explicit permission for the use of their data in academic publications.

### 2.3. Research tools

#### 2.3.1. General information questionnaire

The general information questionnaire collected basic sociodemographic characteristics, including age, sex, marital status, educational level, and type of medical insurance, as well as cancer-related clinical data such as cancer type, clinical stage, chemotherapy regimen, number of chemotherapy cycles, and presence of comorbidities.

#### 2.3.2. Geriatric Depression Scale–15, GDS-15

The Geriatric Depression Scale–15 (GDS-15) was originally developed by Sheikh et al^[19]^ in 1986. Tang et al^[20]^ later validated the psychometric properties of the scale in the Chinese elderly population. The GDS-15 consists of 15 items assessing satisfaction with life, cognitive status, and depressive symptoms. Some items are reverse-scored. Respondents answer each item with “yes” (scored as 1) or “no” (scored as 0), with higher total scores indicating more severe depressive symptoms. The Cronbach α coefficient for the scale was 0.793.

#### 2.3.3. Chemotherapy-induced peripheral neuropathy assessment tool

The chemotherapy-induced peripheral neuropathy assessment tool was originally developed by Professor Tofthagen and colleagues in the United States.^[21]^ In the present study, we used the Chinese version translated and validated by Wang Yue et al.^[18]^ The scale consists of 2 parts, and we adopted the first part to assess patients’ experiences of CIPN symptoms. This section includes 9 symptom items. Some items are scored dichotomously (0 = absent, 1 = present), while others are rated on a scale from 0 to 10. The total score ranges from 0 to 279, with higher scores indicating more severe CIPN symptoms. The Cronbach α coefficient for the scale was 0.880.

#### 2.3.4. Cancer Fatigue Scale, CFS

The CFS was compiled by Okuyama et al,^[22]^ and subsequently translated into Chinese by Zhang Fengling et al.^[23]^ It comprises 15 items covering 3 dimensions: physical fatigue, emotional fatigue, and cognitive fatigue. Each item is rated on a 5-point Likert scale (0–4), with a total score ranging from 0 to 60. A score above 18 indicates the presence of fatigue, with higher scores reflecting greater fatigue severity. The Cronbach α coefficient for the scale was 0.925.

#### 2.3.5. Perceived Social Support Scale

The Perceived Social Support Scale, originally developed by Zimet et al,^[24]^ and later translated and adapted into Chinese by Jiang Qianjin et al.^[25]^ It consists of 11 items encompassing 3 dimensions: support from family, friends, and significant others. Each item is rated on a 7-point Likert scale ranging from 1 (“strongly disagree”) to 7 (“strongly agree”), with total scores ranging from 12 to 84. Higher scores indicate greater levels of perceived social support. The Cronbach α coefficient for the scale was 0.950.

### 2.4. Data collection process

In this study, data were collected through questionnaires combined with medical records review. After obtaining the consent of the director of the oncology center and the signing of the informed consent form by the patients, the uniformly trained investigators distributed the questionnaires to the patients in a “one-on-one, face-to-face” manner and explained the method of filling out the questionnaires. Patients were encouraged to complete the questionnaire independently, and if they had difficulties, they were assisted in completing the questionnaire in a way that avoided guiding hints. The questionnaires were distributed and collected on site, and the clinical information was supplemented by the medical records. A total of 309 questionnaires were distributed and 290 valid questionnaires were recovered, with an effective recovery rate of 93.8%.

### 2.5. Statistical analysis

All data analyses were performed using SPSS version 26.0. The distribution pattern of continuous data was examined using P-P plots. Continuous data conforming to a normal distribution were presented as mean ± standard deviation (SD). For intergroup comparisons of continuous data, the independent samples *t*-test was applied. Categorical data were expressed as percentages, and the chi-square test was utilized for intergroup comparisons. Pearson correlation analysis was used to examine the correlations among the continuous variables of depression, CRF, CIPN, and PSS. Prior to testing the moderated mediation model, collinearity diagnosis was conducted using variance inflation factor (VIF) and Tolerance to ensure that no significant multicollinearity would bias the parameter estimates. Detailed diagnostic results are supplemented in the following sections. The mediation effect model of CIPN in the relationship between depression and CRF was constructed using Model 4 of the PROCESS macro in SPSS, while Model 8 of the same macro was employed to build the moderating effect model of PSS, with 5000 bootstrap resamples to estimate indirect effects and their 95% confidence intervals, The test level α = 0.05.

## 3. Results

### 3.1. Baseline characteristics and univariate analysis of CRF in elderly CRC patients

A total of 290 elderly CRC patients with CIPN were included, of whom 62.8% were male. Most participants were aged 60 to 69 years (57.2%), had a normal BMI (51.0%), and completed junior high school (66.9%). The majority were insured under employee medical insurance (54.5%) and diagnosed with stage IV disease (62.1%). A history of smoking and alcohol consumption was reported in over half of the patients. Stoma was present in 26.2% of participants. Regarding comorbidities, 43.4% had at least one chronic condition. The XELOX regimen was the most commonly used (65.5%), and 39.3% received 6 or more chemotherapy cycles. Detailed characteristics are summarized in Table 1.

**Table 1:** Distribution of demographic and clinical characteristics and univariate analysis of CRF in elderly CRC patients (n = 290).

Variable	Category	N	%	CRF		
				(M ± SD)	*T/Z*	*P*
Age (yr)	60–69	166	57.2	17.00 ± 6.77	12.147	.002
	70–79	110	37.9	20.36 ± 5.93		
	≥80	14	0.7	15.00 ± 4.93		
BMI (kg/m^2^)	<18.5	22	7.6	19.09 ± 6.64	0.225	.947
	18.5–23.9	148	51.0	18.01 ± 6.82		
	24–27.9	116	40.0	18.28 ± 6.35		
	≥28	4	1.4	16.50 ± 12.02		
Sex	Male	182	62.8	18.08 ± 6.93	0.242	.809
	Female	108	37.2	18.35 ± 6.04		
Education level	Primary school or below	70	24.1	18.37 ± 5.30	2.310	.511
	Junior high school	194	66.9	17.76 ± 6.90		
	Senior high school/Technical secondary school	20	6.9	21.80 ± 7.93		
	College or above	6	2.1	17.33 ± 2.52		
Medical insurance type	Urban resident insurance	64	22.1	19.38 ± 5.73	6.490	.261
	Commercial insurance	10	3.4	18.04 ± 6.57		
	Provincial-level insurance	4	1.4	21.20 ± 7.01		
	Rural insurance	26	9.0	19.00 ± 0.61		
	Employee insurance	158	54.5	19.00 ± 7.51		
	Self-paid	28	9.7	14.29 ± 7.26		
Tumor stage	I	2	1.4	17.50 ± 0.71	5.953	.114
	II	30	10.3	15.20 ± 6.14		
	III	76	26.2	17.37 ± 6.12		
	IV	180	62.1	19.03 ± 6.80		
Smoking history	No	134	46.2	17.00 ± 6.78	−2.018	.045
	Yes	156	53.8	19.19 ± 6.29		
Alcohol consumption	No	130	44.8	17.32 ± 6.64	−1.350	.179
	Yes	160	55.2	18.81 ± 6.52		
Stoma presence	No	214	73.8	18.21 ± 6.60	0.080	.936
	Yes	76	26.2	18.11 ± 6.65		
Chronic disease history	No	164	56.6	17.68 ± 6.66	6.230	.101
	Hypertension	66	22.8	17.41 ± 6.31		
	Diabetes	10	3.4	23.00 ± 6.46		
	Both	48	17.2	19.96 ± 6.42		
Chemotherapy regimen	FOLFOX	82	28.3	18.29 ± 6.25	4.796	.091
	XELOX	190	65.5	17.67 ± 6.48		
	Others	18	6.2	23.00 ± 7.97		
Number of chemotherapy cycles	≥6	114	39.3	16.16 ± 5.93	−3.058	.003
	<6	176	60.7	19.49 ± 6.69		

BMI = body mass index, Both = Hypertension+Diabetes, CRF = cancer-related fatigue, FOLFOX = oxaliplatin + leucovorin + 5-fluorouracil, M = mean, SD = standard deviation, XELOX = oxaliplatin + capecitabine; and other regimens such as FOLFOXIRI (irinotecan + oxaliplatin + 5-fluorouracil + leucovorin) or combinations of FOLFOX and XELOX.

* *P* < .05.

** *P* < .001.

Univariate analysis revealed significant differences in CRF levels with respect to age, smoking history, and chemotherapy cycles (*P* < .05). No statistically significant differences were observed for the other variables (as shown in Table 1). Therefore, these 3 variables will be included as covariates in subsequent analyses.

### 3.2. Descriptive and correlational analysis of the key variables in the mediation model

The means and standard deviations of the main variables in this study were as follows: the mean depression score was 6.200 ± 3.683, the PSS score was 41.601 ± 14.290, the total CIPN symptom experience score was 105.331 ± 42.561, and the CRF score was 18.179 ± 6.590. The correlation analysis revealed significant relationships among the variables (*P* < .001), with depression positively correlated with both CRF and CIPN, and PSS negatively correlated with both CIPN and CRF (as shown in Table 2).

**Table 2:** Statistical and Pearson correlation analysis results.

Variable	Basic statistics		The correlation coefficient between variables			
	M	SD	1	2	3	4
Depression	6.200	3.683	1			
PSS	41.601	14.290	−0.106	1		
CIPN	105.331	42.561	0.247**	−0.641**	1	
CRF	18.179	6.590	0.336**	−0.604**	0.573**	1

Abbreviations: CIPN = chemotherapy-induced peripheral neuropathy, CRF = cancer-related fatigue, M = mean, PSS = perceived social support, SD = standard deviation.

* *P* < .05.

** *P* < .001.

### 3.3. Multicollinearity diagnostics

Before conducting the mediation analysis, we performed multicollinearity diagnostics. The results showed that all variance inflation factor values were below 10, and Tolerance values were above 0.1 (as shown in Table 3), indicating that multicollinearity did not pose a significant threat to the model’s parameter estimates.

**Table 3:** Multicollinearity diagnostics of independent variables.

Variable	Tolerance	VIF
Depression	0.914	1.094
PSS	0.573	1.747
CIPN	0.530	1.888
Depression × PSS	0.963	1.038

CIPN = chemotherapy-induced peripheral neuropathy, CRF = cancer-related fatigue, Depression×PSS = the interaction term between depression Depression and PSS, PSS = perceived social support. This interaction term is used to test whether PSS moderates the relationships between depression and CIPN, as well as between depression and CRF, in the moderated mediation model.

### 3.4. The mediating role of CIPN

To examine the potential mediating role of CIPN in the relationship between depression and CRF, we conducted a mediation analysis using Hayes’ PROCESS macro (Model 4). CRF was specified as the dependent variable, depression as the independent variable, and CIPN as the mediating variable. As shown in Table 4, depression was a significant positive predictor of CRF (β = 0.601, *P* < .001). The direct effect of depression on CRF still remained significant after the inclusion of CIPN (β = 0.370, *P* < .05). Additionally, CIPN was a significant positive predictor of CRF (β = 0.081, *P* < .05), indicating that CIPN may play a mediating role between depression and CRF.

**Table 4:** Testing the mediating effect of CIPN.

Dependent variable	Independent variable	Fit index		Coefficient significance		
		*R*	*R^2^*	*F*	*β*	*t*
CIPN	Depression	0.247	0.061	9.270*	2.851	3.045*
CRF	Depression	0.336	0.113	18.147**	0.601	4.260**
CRF	Depression	0.607	0.369	41.456*	0.370	3.005*
	CIPN				0.081	7.589**

CIPN = chemotherapy-induced peripheral neuropathy, CRF = cancer-related fatigue.

* *P *< .05.

** *P *< .001.

To rigorously assess the significance of this mediating effect, we further evaluated it using the Bootstrap method (5000 replicated samples, 95% confidence interval [CI]), revealing a significant mediation effect (as shown in Table 5). The total effect of depression on CRF was 0.601 (95% CI: 0.322–0.879), with a direct effect of 0.370 (95% CI: 0.126–0.613) and an indirect effect of 0.231 (95% CI: 0.087–0.409), with the mediating effect accounting for 38.44% of the total effect. These results suggest that CIPN may partially mediate the relationship between depression and CRF.

**Table 5:** Decomposition table of total effect, direct effect, and indirect effect.

	Point estimate	Standard error	95% CI		Ratio of effect (%)
			Lower	Upper	
Total effect	0.601	0.141	0.322	0.879	100.00
Direct effect	0.370	0.123	0.126	0.613	61.56
Indirect effect	0.231	0.083	0.087	0.409	38.44

CI = confidence intervals.

### 3.5. Moderating mediating effects of PSS

**Figure 1.**
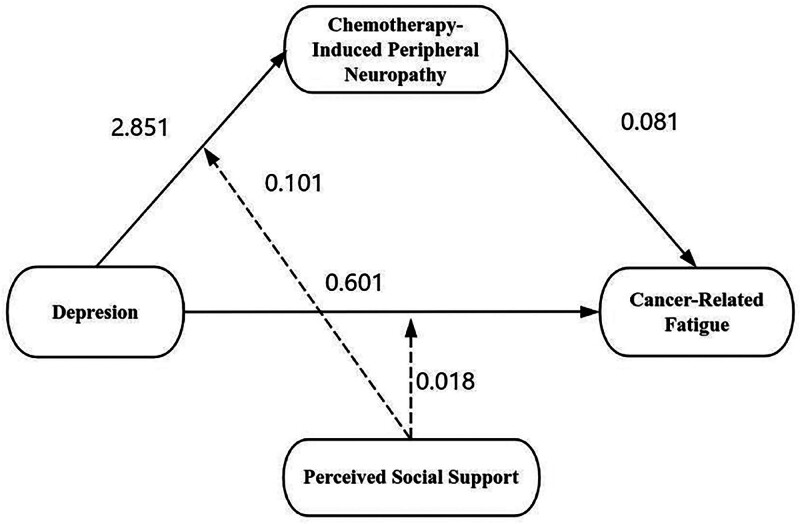
Influence pathways for the moderating role of perceived social supported.

In this study, we employed the PROCESS macro Model 8 to test whether PSS moderates the “depression → CIPN → CRF” pathway. After controlling for relevant covariates, the interaction term depression × PSS significantly predicted both CIPN (β = 0.101, *t* = 2.041, *P* < .05) and CRF (β = 0.018, *t* = 2.387, *P* < .05) (as shown in Table 6). The interaction accounted for 47.0% and 51.7% of the variance in the respective models. These findings suggest that PSS exerts a positive moderating effect on both the “depression → CIPN” and “depression → CRF” pathways, indicating that higher levels of perceived social support strengthen the associations between depression and both CIPN and CRF, as illustrated in Figure 1.

**Table 6:** Path-coefficients of the moderated mediating model.

Dependent variable	Independent variable	Fit index		Coefficient significance		
		*R*	*R^2^*	*F*	*β*	*t*
CIPN	Depression × PSS	0.686	0.470	4.167*	0.101	2.041*
CRF	Depression × PSS	0.719	0.517	5.696*	0.018	2.387*

CIPN = chemotherapy-induced peripheral neuropathy, CRF = cancer-related fatigue, Depression×PSS = the interaction term between depression and PSS, PSS = perceived social support.

* *P* < .05.

** *P* < .001.

**Figure 2.**
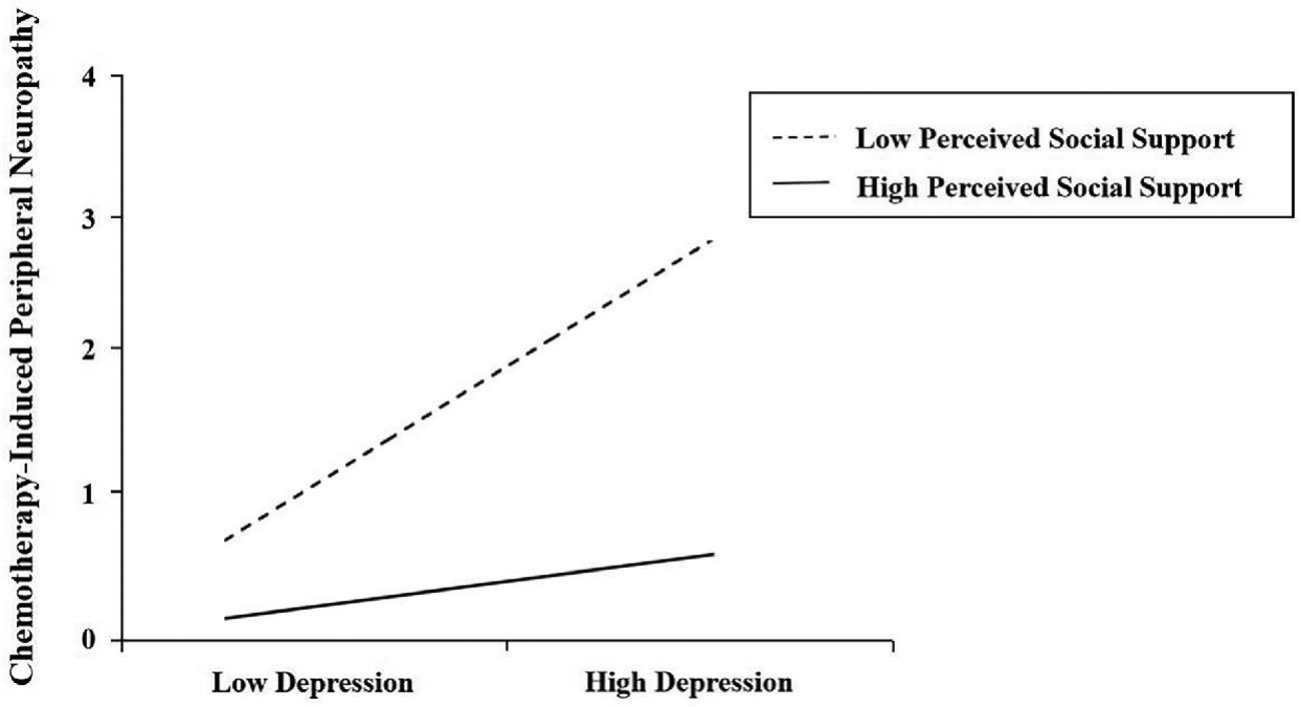
The moderating effect of perceived social support on the relationship between depression and chemotherapy-induced peripheral neuropathy.

To further examine the moderating effect of PSS, PSS levels were categorized into low, medium, and high based on the mean ± 1 standard deviation, and simple slope analyses were conducted, as shown in Table 7. As shown in Figure 2, the relationship between depression and CIPN was not significant in the low-PSS group (simple slope = 0.7776, 95% CI: −1.2501 to 2.8052). However, this association became significant in the medium-PSS group (simple slope = 2.2161, 95% *CI*: 0.8532–3.5790) and the effect was further enhanced in the high-PSS group (simple slope = 3.6546, 95% *CI*: 1.7874–5.5219).

**Table 7:** Analysis of the regulatory role of perceived social supported.

Path	PSS	Effect	Boot SE	95% CI	
				Lower	Upper
Depression-CIPN	−14.2898 (M-1SD)	0.7776	1.0255	−1.2501	2.8052
	0.0000 (M)	2.2161	0.6893	0.8532	3.5790
	14.2898 (M + 1SD)	3.6546	0.9444	1.7874	5.5219
Depression-CRF	−14.2898 (M-1SD)	0.0899	0.1587	−0.2238	0.4037
	0.0000 (M)	0.3535	0.1103	0.1354	0.5717
	14.2898 (M + 1SD)	0.6171	0.1535	0.3136	0.9206

CIPN = chemotherapy-induced peripheral neuropathy, CRF = cancer-related fatigue, M = mean, PSS = perceived social support, SD = standard deviation.

**Figure 3.**
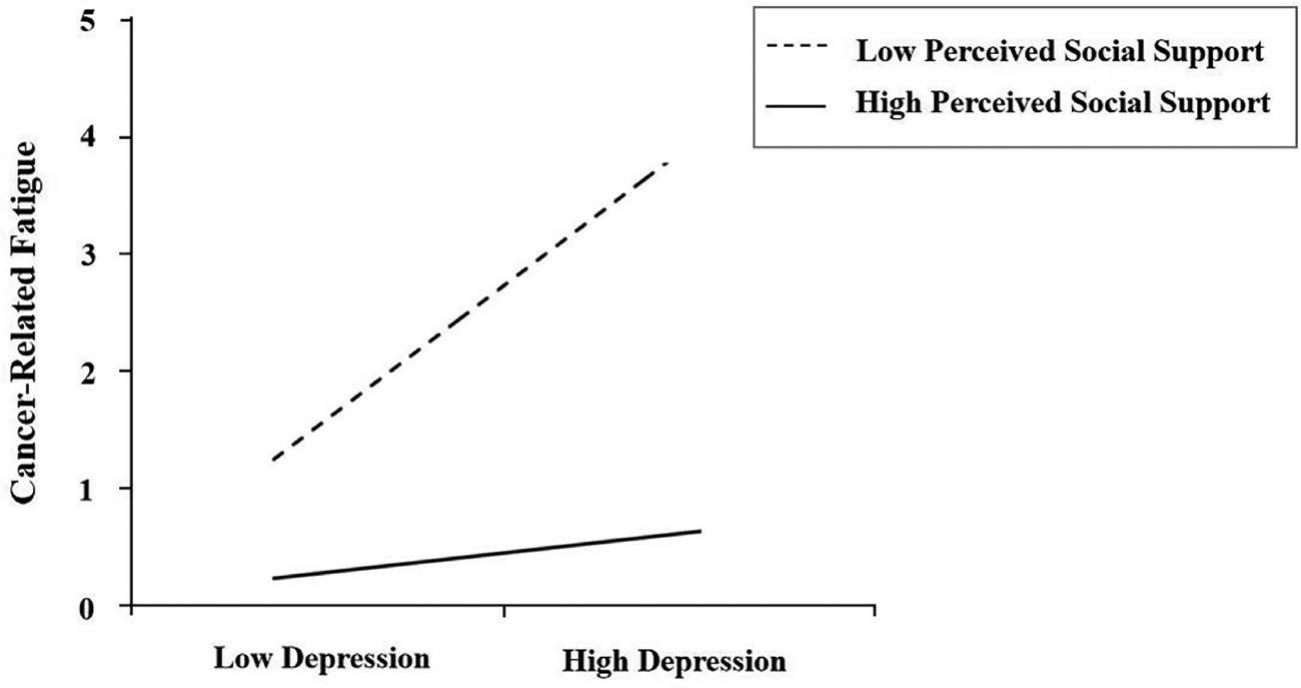
The moderating effect of perceived social support on the relationship between depression and cancer-related fatigue.

Similarly, as shown in Figure 3, the effect of depression on CRF was not significant in the low-PSS group (simple slope = 0.0899, 95% *CI*: −0.2238–0.4037), but was significant in the medium-PSS group (simple slope = 0.3535, 95% CI: 0.1354–0.5717), and became even stronger in the high-PSS group (simple slope = 0.6171, 95% CI: 0.3136–0.9206). In summary, these results consistently demonstrate that higher levels of perceived social support amplify the positive relationships between depression and both CIPN and CRF, indicating a moderating pattern that diverges from the conventional stress-buffering hypothesis.

## 4. Discussion

### 4.1. Correlation of depression and CRF during chemotherapy in elderly CRC patients

The present study further confirmed that depression was significantly and positively associated with CRF in elderly patients with colorectal cancer, consistent with the findings of Jolly et al.^[26]^ As a typical manifestation of chronic stress response, depression is often accompanied by sleep disturbances, anhedonia, and negative cognitive biases, all of which may be linked to reduced physical resilience and motivation, thereby inducing or aggravating persistent fatigue.^[10]^ Additionally, elderly CRC patients frequently face multiple physical burdens and functional decline, making them more vulnerable to the adverse effects of depression on coping capacity,^[27]^ which may further perpetuate the fatigue-depression cycle. These findings suggest that CRF is not only a direct side effect of chemotherapy, but also may reflect the interplay between cumulative psychological stress and physiological systems. Therefore, routine clinical care should emphasize emotional assessment and psychological support, regard depression as a key target in fatigue management, and develop culturally tailored, individualized interventions to enhance quality of life and support recovery.

### 4.2. The mediating role of CIPN between depression and CRF

After adjusting for relevant covariates, CIPN symptoms were found to partially mediate the association between depression and CRF (indirect effect = 0.231, accounting for 38.44% of the total effect). This result not only confirms the indirect pathway through which depression influences CRF, but also, for the first time in elderly Chinese CRC patients, identifies CIPN, a subjectively perceived symptom, as a mediator linking mood and CRF. While cross-sectional, this analysis provides valuable theoretical insights and points to potential clinical implications, which should be further examined in longitudinal studies.

This phenomenon may be closely related to the subjective nature of CIPN assessment. As a chemotherapy-related neurotoxic effect, CIPN is primarily evaluated through patient self-reports, which can be influenced by negative cognitive emotions such as depression. Depression has been linked to disruptions in hypothalamic-pituitary-adrenal axis function and autonomic homeostasis via persistently activating the stress response, which in turn enhances inflammatory responses and neural sensitivity.^[28]^ These physiological changes are associated with heightened attention to and the perception of CIPN-related somatic discomfort. Patients with depression are more likely to focus on physical discomfort, amplifying their perceptual response and thereby intensifying the psychological experience of CIPN.^[8,11]^

In addition, the physical discomfort associated with CIPN can restrict patients’ daily activities and lead to exercise avoidance behaviors.^[5]^ While regular physical activity is one of the most effective non-pharmacological strategies for managing cancer-related fatigue, it has been shown to alleviate CRF significantly by modulating inflammation, activating neuroimmune pathways, and improving mood. However, reduced exercise participation is associated with exacerbation of CRF, resulting in a decline in functional capacity and overall physical resilience.^[29]^

Therefore, healthcare professionals should not only focus on the physiological mechanisms of neurotoxicity, but also recognize the potential influence of psychological factors, such as depression, in shaping the subjective experience of CIPN. By identifying depression early, enhancing psychological support, and promoting safe and feasible physical activities, it is possible to reduce patients’ exaggerated perceptions of CIPN, thereby mitigating its indirect effect on CRF and optimizing the overall rehabilitation pathway for elderly CRC patients.

### 4.3. The moderating role of PSS in mediation models

In this study, we found that PSS positively moderated both the indirect pathway from depression to CRF via CIPN and the direct pathway from depression to CRF. Interestingly, higher levels of PSS were associated with stronger positive associations between depression and both CIPN and CRF. This finding contradicts the traditional buffering hypothesis, suggesting that social support may not always play a protective role in certain populations.

This unexpected phenomenon may be closely associated with the socio-cultural characteristics of the study population. Specifically, 91.0% of participants in this study had an educational level of high school or below and generally lacked systematic health education and emotional coping skills.^[30]^ In this context, individuals may struggle to internalize perceived social support as a positive emotional resource.^[31]^ In particular, under the influence of traditional Chinese cultural values, elderly patients tend to perceive their illness as a burden to their families.^[32]^ Thus, well-intentioned support from family members might be reinterpreted by elderly patients as a reminder of their perceived burden and failure to fulfill family roles, thereby exacerbating emotional distress.^[33,34]^ Elderly patients with CRC are prone to elevated self-stigma,^[35]^ In such a psychological context, increased social support may be positively associated with heightened levels of anxiety and depression.^[36]^

Furthermore, the specific form and quality of support is expressed may be just as important as its presence. When support takes the form of overprotection or constant focus on the patient’s symptoms, which can inadvertently intensify their focus on physical discomfort.^[12]^ This heightened symptom vigilance may amplify the subjective experience of CIPN, subsequently resulting in movement avoidance and functional impairment.^[5]^ These consequences can hinder physical recovery, reduce perceived energy levels, and ultimately worsen CRF. Under chronic caregiving pressure, interpersonal friction or the patient’s deep sense of guilt may cause support behaviors to be perceived as “obligatory effort” rather than genuine care, which can aggravate psychological distress and indirectly exacerbates somatic symptoms.^[37]^

Finally, methodological constraints may also have contributed to the observed amplification effect. Given the cross-sectional design of this study, reverse causality cannot be ruled out, it is possible that patients with stronger depressive tendencies, due to their negative cognitive bias, are more likely to perceive all forms of support as burdensome rather than beneficial.^[38]^ Moreover, the PSS scale used in this study did not differentiate between support types or relationship quality, limiting our ability to identify which forms of support might be maladaptive. Future research should employ refined measurements to verify these interaction mechanisms and clarify the dynamic interplay between emotional states and perceived support.

In summary, the impact of PSS is not universally beneficial but may be shaped by a combination of cultural values, types of support, and individuals’ cognitive interpretations. For elderly Chinese CRC patients, high levels of perceived support may paradoxically coincide with increased symptom awareness and distress.

Therefore, clinical interventions should emphasize the individualization and contextual adaptation of support strategies. Attention should be paid not only to the quantity of support provided, but also to patients’ subjective acceptance and psychological responses. Future intervention designs should incorporate cultural sensitivity assessments and psychological response monitoring to enhance the effectiveness of social support.

## 5. Conclusion

This study explored a moderated mediation model to examine the interrelationships among depression, CIPN, CRF, and PSS in elderly Chinese patients with CRC. The results demonstrated that CIPN symptoms partially mediated the relationship between depression and CRF. Furthermore, PSS significantly moderated the pathways from depression to both CIPN and CRF, strengthening rather than buffering these relationships. Contrary to the conventional stress-buffering hypothesis, these findings suggest that perceived social support does not uniformly function as a protective factor in this population.

Overall, the study enhances the theoretical understanding of psychological mechanisms underlying tumor-related symptoms and offers empirical evidence to guide emotional interventions and symptom management in elderly CRC patients. Future clinical practice should emphasize identifying and addressing the interactions between psychological factors and subjective symptoms, with particular attention to the cultural and contextual appropriateness of support delivery and patients’ emotional receptivity, in order to provide more individualized and culturally sensitive care.

## 6. Limitations

This study has several limitations. Due to the cross-sectional design, causal relationships between variables cannot be established. Future longitudinal studies or intervention trials are warranted to verify the stability and directionality of the proposed model. All core variables, depression, CIPN, CRF, and perceived social support, were assessed using self-report measures. Although this approach captures patients’ subjective experiences, it introduces the risk of common method variance and potential symptom overlap across constructs (e.g., between depression and fatigue, or between depressive cognitions and CIPN perceptions). The reliance on self-report also increases susceptibility to cognitive biases and social desirability effects, particularly among elderly participants. To enhance the validity of future findings, it would be beneficial to incorporate objective or clinician-rated indicators, such as standardized neuropathy assessments, biomarkers of inflammation, or actigraphy-based measures of fatigue and activity. The sample was drawn from a single tertiary hospital with a high proportion of participants with lower educational attainment, which may limit the generalizability of the findings to broader populations. Additionally, other potentially influential variables such as inflammation markers, sleep quality, and comorbidity severity were not included. Future research should incorporate and control for these potential confounders to enhance the model’s explanatory power.

## Acknowledgments

We express our gratitude to all staff members of the affiliated hospital of Jiangnan University.

## Author contributions

**Conceptualization:** Wenjing Xu, Qian Cao.

**Data curation:** Chen Xu, Jinyou Song.

**Formal analysis:** Chen Xu, Jinyou Song.

**Writing – original draft:** Qian Cao.

**Writing – review & editing:** Yuling Yang, Ying Chen.

## References

[1] Morgan E Arnold M Gini A. Global burden of colorectal cancer in 2020 and 2040: incidence and mortality estimates from GLOBOCAN. Gut. 2023;72:338–44. 36604116 10.1136/gutjnl-2022-327736

[2] Li Q Wu H Cao M. Colorectal cancer burden, trends and risk factors in China: a review and comparison with the United States. Chin J Cancer Res. 2022;34:483–95. 36398126 10.21147/j.issn.1000-9604.2022.05.08 PMC9646460

[3] O’Donnell CDJ Hubbard J Jin Z. Updates on the management of colorectal cancer in older adults. Cancers (Basel). 2024;16:1820. 38791899 10.3390/cancers16101820 PMC11120096

[4] Cheng F Zhang R Sun C. Oxaliplatin-induced peripheral neurotoxicity in colorectal cancer patients: mechanisms, pharmacokinetics and strategies. Front Pharmacol. 2023;14:1231401. 37593174 10.3389/fphar.2023.1231401 PMC10427877

[5] Hines RB Schoborg C Sumner T Thiesfeldt DL Zhang S. The associations of oxaliplatin-induced peripheral neuropathy, sociodemographic characteristics, and clinical characteristics with time to fall in older adults with colorectal cancer. Am J Epidemiol. 2024;193:1271–80. 38751324 10.1093/aje/kwae067 PMC11483325

[6] Vlaski T Slavic M Caspari R Fischer H Brenner H Schöttker B. Development trajectories of fatigue, quality of life, and the ability to work among colorectal cancer patients in the first year after rehabilitation-first results of the MIRANDA study. Cancers (Basel). 2023;15:3168. 37370777 10.3390/cancers15123168 PMC10296005

[7] Ee C Kay S Reynolds A Lovato N Lacey J Koczwara B. Lifestyle and integrative oncology interventions for cancer-related fatigue and sleep disturbances. Maturitas. 2024;187:108056. 38981156 10.1016/j.maturitas.2024.108056

[8] Schwab L Visovsky C. Psychological distress and quality of life in breast cancer survivors with taxane-induced peripheral neuropathy: a scoping review. Front Oncol. 2022;12:1005083. 36703798 10.3389/fonc.2022.1005083 PMC9872004

[9] Hsiao CP Von Ah D Chen MK Saligan LN. Relationship of cancer-related fatigue with psychoneurophysiological (PNP) symptoms in breast cancer survivors. Eur J Oncol Nurs. 2024;68:102469. 38039708 10.1016/j.ejon.2023.102469 PMC10922833

[10] Balakin E, Turku K Ivanov M Izotov A Nakhod V Pustovoyt V. Regulation of stress-induced immunosuppression in the context of neuroendocrine, cytokine, and cellular processes. Biology (Basel). 2025;14:76. 39857306 10.3390/biology14010076 PMC11760489

[11] Zhang C Wang J He Y. Depression impact on PTSD in Cancer patients through serial mediation of hope and perceived social support. Sci Rep. 2025;15:24727. 40634464 10.1038/s41598-025-09908-w PMC12241477

[12] Hughes S Jaremka LM Alfano CM. Social support predicts inflammation, pain, and depressive symptoms: longitudinal relationships among breast cancer survivors. Psychoneuroendocrinology. 2014;42:38–44. 24636499 10.1016/j.psyneuen.2013.12.016 PMC3970938

[13] Castarlenas E Galán S Solé E. Perceived stress, perceived social support, and global health in adults with chronic pain. Int J Behav Med. 2025;32:92–101. 38129718 10.1007/s12529-023-10250-6 PMC11790680

[14] Williams GR Pisu M Rocque GB. Unmet social support needs among older adults with cancer. Cancer. 2019;125:473–81. 30508291 10.1002/cncr.31809

[15] Flores NJ Mathew MJ Fortson LS Abernethy AD Ashing KT. The influence of culture, social, and religious support on well-being in breast cancer survivorship. Cureus. 2021;13:e14158. 33936871 10.7759/cureus.14158 PMC8078221

[16] Harris RJ. A primer of multivariate statistics. Psychology Press; 2001.

[17] Sim M Kim SY Suh Y. Sample size requirements for simple and complex mediation models. Educ Psychol Meas. 2022;82:76–106. 34992307 10.1177/00131644211003261 PMC8725051

[18] Wang Y Fu JF Bai YN Xie J Jiao HX Wang ZL. A survey on nurses’ knowledge, attitudes, and practices regarding the assessment and management of chemotherapy - induced peripheral neuropathy. Chin Nurs Manag. 2018;18:382–7.

[19] Cruice M Worrall L Hickson L. Reporting on psychological well-being of older adults with chronic aphasia in the context of unaffected peers. Disabil Rehabil. 2011;33:219–28. 20629580 10.3109/09638288.2010.503835

[20] Tang D. Application of the short-form Geriatric Depression Scale (GDS-15) among Chinese older adults. Chin J Clin Psychol. 2013;21:402–5.

[21] Tofthagen CS McMillan SC Kip KE. Development and psychometric evaluation of the chemotherapy-induced peripheral neuropathy assessment tool. Cancer Nurs. 2011;34:E10–20. 10.1097/NCC.0b013e31820251de 21242773

[22] Okuyama T Akechi T Kugaya A. Factors correlated with fatigue in disease-free breast cancer patients: application of the Cancer Fatigue Scale. Support Care Cancer. 2000;8:215–22. 10789963 10.1007/s005200050288

[23] Zhang F Ding Y Han LS. Reliability and validity of the Chinese version of the Cancer Fatigue Scale. Chin Mental Health J. 2011;25:810–3.

[24] Zimet GD Powell SS Farley GK Werkman S Berkoff KA. Psychometric characteristics of the multidimensional scale of perceived social support. J Pers Assess. 1990;55:610–7. 2280326 10.1080/00223891.1990.9674095

[25] Zhang F Zhu SZ Deng PJ. Evaluation of the perceived social support scale in hospitalized patients in China. Nurs Res. 2018;32:2048–52.

[26] Jolly AA Anyanwu S Koohi F Morris RG Markus HS. Prevalence of fatigue and associations with depression and cognitive impairment in patients with CADASIL. Neurology. 2025;104:e213335. 39819095 10.1212/WNL.0000000000213335 PMC11737844

[27] Ruiz-Casado A Álvarez-Bustos A de Pedro CG Méndez-Otero M Romero-Elías M. Cancer-related fatigue in breast cancer survivors: a review. Clin Breast Cancer. 2021;21:10–25. 32819836 10.1016/j.clbc.2020.07.011

[28] Lei AA Phang WWX Lee YZ. Chronic stress-associated depressive disorders: the impact of HPA axis dysregulation and neuroinflammation on the hippocampus-a mini review. Int J Mol Sci . 2025;26:2940. 40243556 10.3390/ijms26072940 PMC11988747

[29] LaVoy EC Fagundes CP Dantzer R. Exercise, inflammation, and fatigue in cancer survivors. Exerc Immunol Rev. 2016;22:82–93. 26853557 PMC4755327

[30] Corovic S Vucic V Mihaljevic O. Social support score in patients with malignant diseases-with sociodemographic and medical characteristics. Front Psychol. 2023;14:1160020. 37325739 10.3389/fpsyg.2023.1160020 PMC10267316

[31] Liu Z Zhao X Zhao L Zhang L. Relationship between perceived social support and mental health among Chinese college football athletes: a moderated mediation model. BMC Psychol. 2023;11:329. 37822005 10.1186/s40359-023-01357-2 PMC10568796

[32] Krause N Liang J. Stress, social support, and psychological distress among the Chinese elderly. J Gerontol. 1993;48:P282–91. 8228001 10.1093/geronj/48.6.p282

[33] Luo Z Zhong S Zheng S. Influence of social support on subjective well-being of patients with chronic diseases in China: chain-mediating effect of self-efficacy and perceived stress. Front Public Health. 2023;11:1184711. 37427286 10.3389/fpubh.2023.1184711 PMC10325675

[34] Krause N Liang J Gu S. Financial strain, received support, anticipated support, and depressive symptoms in the People’s Republic of China. Psychol Aging. 1998;13:58–68. 9533190 10.1037//0882-7974.13.1.58

[35] Xi Z Rong CM Ling LJ. The influence of stigma and disability acceptance on psychosocial adaptation in patients with stoma: a multicenter cross-sectional study. Front Psychol. 2022;13:937374. 36571011 10.3389/fpsyg.2022.937374 PMC9773876

[36] Amin SM Khedr MA Tawfik AF Gamal Noaman Malek M El-Ashry AM. The mediating and moderating role of social support on the relationship between psychological well-being and burdensomeness among elderly with chronic illness: community nursing perspective. BMC Nurs. 2025;24:156. 39930516 10.1186/s12912-025-02743-4 PMC11812208

[37] Chen X Wang Z Zhou J Loke AY Li Q. A scoping literature review of factors influencing cancer patients’ self-perceived burden. Eur J Oncol Nurs. 2024;68:102462. 37995428 10.1016/j.ejon.2023.102462

[38] Keeler AR Nydegger LA Crano WD. Combatting negative bias: a mental contrasting and implementation intentions online intervention to increase help-seeking among individuals with elevated depressive symptomatology. Front Psychol. 2023;14:1145969. 37397325 10.3389/fpsyg.2023.1145969 PMC10310967

